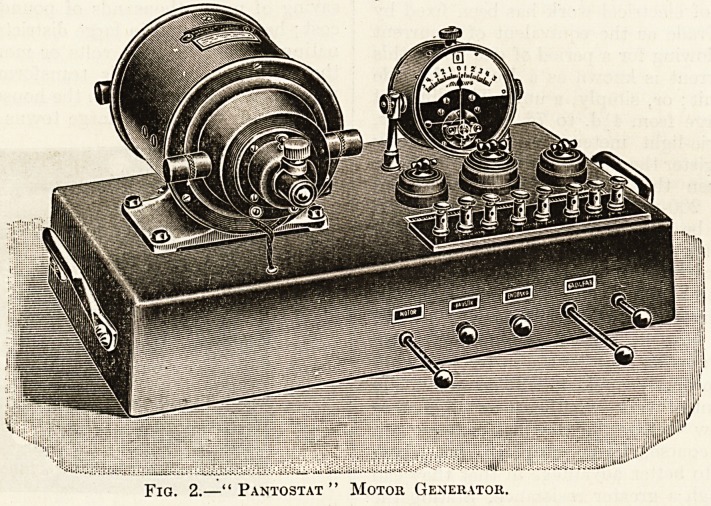# Current from the Main

**Published:** 1912-01-27

**Authors:** Alfred C. Norman

**Affiliations:** House Surgeon at Sunderland and Durham County Eye Infirmary.


### ELECTRICITY IN MODERN MEDICINE.*

### VI.—Current from the Main.

By ALFRED C. NORMAN, M.D. Edin., House Surgeon at Sunderland and Durham County Eye Infirmary.

It is advisable at this stage to consider some technical points in connection with commercial electric lighting, for a knowledge of these will be useful when we come to adapt current from the main to electro-medical requirements.

### Some Technical Points.

The watt is the practical unit of electrical power; it is equivalent to a current of 1 ampère discharged at a pressure of 1 volt, and is capable of doing one seven-hundred-and-forty-sixth horse-power of work in a second. A larger unit of power is the kilowatt, which is equal to 1000 watts. For commercial purposes the unit of electrical work has been fixed by the Board of Trade as the equivalent of a current of 1000 watts flowing for a period of one hour; this quantity of current is known as a Board of Trade unit; a B.T. unit; or, simply, a unit. The different companies charge from $4\frac{1}{2}$d. to 7d. per B.T. unit, and all electric-light meters are now directly graduated to register the number of units consumed.

We have seen that watts = ampères × volts; therefore, on a 200-volt supply a unit (1000 watt-hours) would be represented by a current of 5 ampères flowing for one hour, whereas on a 100-volt supply a current of 10 ampères for one hour would constitute a unit. (Of course a correspondingly larger current, flowing for a shorter period, would in each case be equivalent to a unit, and would be registered as such by the meter.) A unit of current, if properly applied, will do identically the same amount of work, whether it be supplied at a high or a low voltage; in the high-voltage unit there are, of course, fewer ampères, but these ampères work to better advantage in that they can be forced through a greater resistance. Stating the above facts in another way, we may say that the lower the voltage of the supply the greater will be the ampèrage required to do a given amount of work, and *vice versa*.

### A Practical Example.

In the following example, work done by 56 watts of current is represented by the production of 16 candle-power of light: On a 224-volt supply we find that a 16 candle-power lamp consumes $\frac{1}{4}$ ampère of current, whereas on a 112-volt supply a 16 candle-power lamp uses $\frac{1}{2}$ ampère, but the total number of watts consumed is the same in each case —namely, 56 (for $\frac{1}{4}$ ampère by 224 volts = 56 watts; and $\frac{1}{2}$ ampère by 112 volts = 56 watts). This result is due to the fact that the lamps are specially made to suit the respective voltages: the filament of the 224-volt lamp is longer and thinner (*i.e.* of greater resistance) than that of the 112-volt lamp, consequently it lets through less current—$\frac{1}{4}$, as compared with $\frac{1}{2}$ ampère; but the $\frac{1}{4}$ ampère produces as much light in traversing the long thin filament of the 224-volt lamp as does the $\frac{1}{2}$ ampère in passing through the shorter and thicker filament of the 112-volt lamp.

The advantage to the electric lighting companies in supplying current at a high voltage lies in the fact that smaller cables can be used to carry a given number of watts; for, the higher the voltage the smaller will be the number of ampères per 1000 watts, and it is ampèrage that heats a conductor, thus limiting its carrying capacity. When current has to be carried long distances a reduction in the size of the copper cables employed may effect a saving of many thousands of pounds on their first cost; hence we find in large districts that an alternating current of 1000 volts or more is carried in the street mains, and is transformed to a lower voltage before being used in the houses (see previous section); in moderately large towns the continuous

Fig. 1.—Alternating-Current Transformer.

current of 200 to 250 volts is commonly supplied; while in small towns, where cables are short and their first cost not so serious an item, a continuous current of 100 volts is sometimes installed.

### Why Consumers Prefer Low Voltage.

To consumers the disadvantages of a high voltage are: a greater risk to life in the event of accidental contact with bare wires; the necessity for better insulation to prevent leakage and short-circuits; and the fact that high-voltage lamps are more fragile on account of their long, thin filaments.

Electric House-Lighting.—Two cables, joined in parallel with the street mains, are brought into the basement of the house and are there connected with the meter and main fuses (a main switch is usually inserted here as well, so that when necessary the current may be cut off from the whole installation). From this point a pair of house-cables are carried to a distributing-board, situated at some

* Previous articles in this series have appeared in The Hospital of Nov. 11 and 25, Dec. 9 and 30, and Jan. 13.

fairly central part of the building, and are there connected with two elongated copper plates. From these plates (which, on a continuous-current supply, are positive and negative respectively) pairs of well-insulated wires are carried to different parts of the building, each pair being known as a circuit.

Should a short-circuit occur between any pair of wires they would very quickly become dangerously hot, therefore, as a safeguard against fire, it is the rule to put at least two fuses in every circuit, one on the positive and one on the negative side, and these are conveniently placed on the distributing-board, forming connecting links between the copper plates and the circuit-wires. A fuse consists of a piece of wire made of some alloy of low melting-point; its function being to melt, and thus cut off the current, when the ampèrage flowing in any circuit is greater than the wires will safely carry. On house-circuits fuses are usually arranged to "blow" at 5 ampères; main fuses being set at 20 ampères and upwards.

### Ratio of Lamps to Circuits.

The number of circuits in a building will depend upon the number of lamps to be supplied and the general plan of the rooms and corridors; the usual arrangement being to have from six to twelve lamps on a circuit. Let us take the case of a circuit intended to supply, say, five rooms leading off a single corridor. The two circuit-wires would be carried straight down the corridor without a break (their free ends of course not being allowed to come into contact with each other), and, opposite the entrance to each room, two feed-wires would be soldered to the circuit-wires, the resulting secondary circuit being carried into the room to supply the lamp.

The above arrangement is known as wiring in parallel; it has the advantages that many or few lamps can be put in a circuit, that additional ones can be easily added from time to time without interfering with the others, and that every lamp is independent of its neighbours. In the old series method of wiring, current passed out of one lamp into the next, and from that into a third, and so on, the result being that they all had to be switched on together and that they all went out together if one lamp happened to break. Series-wiring still has its uses where arc-lamps are used for street-lighting.

### Using House Currents for Medical Purposes.

We have now to discuss the various ways of utilising the house-current for medical purposes, and this is best done under two headings: "Continuous Current" and "Alternating Current."

Alternating Current.—Two wires connected with an alternating-current supply become positive and negative alternately in synchronism with the periodicity of the supply, hence the use of this current is restricted to those purposes for which it is not essential to differentiate between the positive and negative poles of the supply. For instance, we can use the alternating current for cautery and light and for a form of faradisation, but it cannot be directly applied for galvanisation, electrolysis, or the production of $x$-rays. It has one advantage over the continuous current (for certain purposes) which is that it can be so easily transformed to a lower voltage. Fig. 1 shows an alternating-current transformer, which can be used to convert a high-voltage alternating current to a lower one suitable for cautery purposes, and for lighting surgical lamps; the addition of rheostats rendering the transformed current absolutely under control.

The most economical method of adapting the alternating current for galvanisation, electrolysis, and ionic medication, is to use a motor-generator. This instrument consists of an alternating-current motor, coupled with a continuous-current dynamo, and will generate a continuous current of any voltage for which the dynamo may be wound. Fig. 2 shows the general arrangement of a motor generator which can be used for many purposes; it is known as the "Pantostat," and will be referred to again in subsequent sections.

*(To be continued.)*

## Figures and Tables

**Fig. 1. f1:**
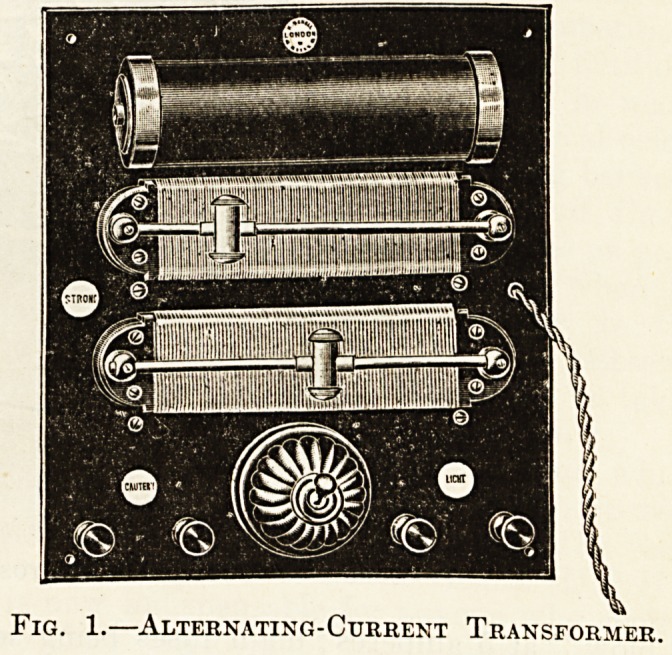


**Fig. 2. f2:**